# Evaluation of Microbiome Alterations Following Consumption of BIOHM, a Novel Probiotic

**DOI:** 10.3390/cimb43030148

**Published:** 2021-11-29

**Authors:** Mahmoud A. Ghannoum, Thomas S. McCormick, Mauricio Retuerto, Gurkan Bebek, Susan Cousineau, Lynn Hartman, Charles Barth, Kory Schrom

**Affiliations:** 1 Department of Dermatology, Case Western Reserve University, Cleveland, OH 44106, USA; tsm4@case.edu (T.S.M.); mauricio.retuerto@case.edu (M.R.); 2 University Hospitals Cleveland Medical Center, Cleveland, OH 44106, USA; Kory.Schrom@uhhospitals.or; 3 Department of Nutrition, Case Western Reserve University, Cleveland, OH 44106, USA; gurkan@case.edu; 4 Fermentation Festival, Santa Barbara, CA 93117, USA; connect@thefarmecologist.com (S.C.); vineescape@gmail.com (L.H.); charlesbartht@gmail.com (C.B.)

**Keywords:** probiotics, dysbiosis, microbiome, mycobiome, *Candida*

## Abstract

Gastrointestinal microbiome dysbiosis may result in harmful effects on the host, including those caused by inflammatory bowel diseases (IBD). The novel probiotic BIOHM, consisting of *Bifidobacterium breve, Saccharomyces boulardii*, *Lactobacillus acidophilus*, *L. rhamnosus*, and amylase, was developed to rebalance the bacterial–fungal gut microbiome, with the goal of reducing inflammation and maintaining a healthy gut population. To test the effect of BIOHM on human subjects, we enrolled a cohort of 49 volunteers in collaboration with the Fermentation Festival group (Santa Barbara, CA, USA). The profiles of gut bacterial and fungal communities were assessed via stool samples collected at baseline and following 4 weeks of once-a-day BIOHM consumption. Mycobiome analysis following probiotic consumption revealed an increase in Ascomycota levels in enrolled individuals and a reduction in Zygomycota levels (*p* value < 0.01). No statistically significant difference in Basidiomycota was detected between pre- and post-BIOHM samples and control abundance profiles (*p* > 0.05). BIOHM consumption led to a significant reduction in the abundance of *Candida* genus in tested subjects (*p* value < 0.013), while the abundance of *C. albicans* also trended lower than before BIOHM use, albeit not reaching statistical significance. A reduction in the abundance of Firmicutes at the phylum level was observed following BIOHM use, which approached levels reported for control individuals reported in the Human Microbiome Project data. The preliminary results from this clinical study suggest that BIOHM is capable of significantly rebalancing the bacteriome and mycobiome in the gut of healthy individuals, suggesting that further trials examining the utility of the BIOHM probiotic in individuals with gastrointestinal symptoms, where dysbiosis is considered a source driving pathogenesis, are warranted.

## 1. Introduction

Human gastrointestinal (GI) microbiome research has primarily focused on resident bacteria and their associated bacterial–host interactions, both beneficial and detrimental. However, solely focusing on bacteria has neglected the potential influence of the host’s fungal community (mycobiome) on health and disease. In a previous study, we characterized the gut bacterial microbiota (bacteriome) and the mycobiome in family members with Crohn’s disease (CD) and their healthy relatives in an attempt to define the interactions leading to dysbiosis in CD. We identified a positive correlation between bacteria and fungi, wherein the bacteria, *Escherichia coli* and *Serratia marcescens*, and the fungus, *Candida tropicalis*, demonstrated increased abundance in the GI tract of CD patients when compared with their non-Crohn healthy relatives [1]. Subsequently, we showed that *C. tropicalis* and the two bacterial species cooperate in a strategic way to form in vitro pathogenic biofilms capable of causing damage to the epithelial cell lining of the gut and initiating an inflammatory response [2]. Not only do these findings identify a possible new therapeutic targeting approach (i.e., bacterial–fungal interaction modulation) in patients with inflammatory bowel disease (IBD), they also highlight a possible avenue for improving human health and disease as a whole through microbiome modulation.

One approach to combat IBD symptoms by preventing and treating microbiome dysbiosis includes the use of probiotics, which the World Health Organization (WHO) has defined as live microorganisms that confer health benefits on the host when administered in adequate amounts [3]. The importance of research and development of probiotics for use in IBD is highlighted in a review by Sartor [4], who reported previously that minimal research has been carried out on probiotics in the setting of IBD, and studies that have been conducted are in relatively small trials with a low number of enrolled patients. Although the numbers of probiotic trials designed to address IBD have increased exponentially, modest cohort size and outcomes still hamper interpretation and limit the rigor of this research [5]. Clearly, there is a need for more clinical trials involving larger numbers of subjects powered sufficiently to statistically address the efficacy of probiotics in GI diseases.

Since the cooperative interaction of fungi and bacteria in the dysbiotic state has been shown to produce harmful effects on the host, it is logical to suggest that the introduction of different combinations of microbes in the form of probiotics to restore overall balance may help to counteract these detrimental effects. Probiotics have been shown to be effective in preventing and ameliorating various medical conditions, particularly those involving the GI tract in children. Recently, certain probiotic bacteria have been studied as a potential method to prevent opportunistic infectious diseases by stimulating the host immune system [6,7,8]. Previous studies have reported the positive effects of probiotics in a variety of diseases such as *Candida* vaginitis [9] and vulvovaginal candidiasis [10,11], oral candidiasis [12], GI infection [13], colon carcinoma [14], and recent probiotic studies on IBD [15,16,17,18,19,20,21,22].

Since it has been demonstrated that microbial dysbiosis is implicated in GI diseases such as IBD, ulcerative colitis, and CD, developing probiotics that can rebalance and maintain the gut microbiota is a reasonable approach to counteract the effect of dysbiosis. The development of the BIOHM probiotic was guided by microbiome analysis based on a large cohort of individuals who were analyzed through the BIOHM gut testing platform to design a probiotic that would affect organisms increased in individuals with intestinal dysbiosis. Our aim was to select appropriate microbes that target pathogenic bacterial and fungal strains while supporting beneficial ones. To achieve this, we conducted correlation analyses of bacterial–bacterial and bacterial–fungal interactions to identify appropriate probiotic strains. This work led to the development of a new probiotic, BIOHM, consisting of *Bifidobacterium breve 19bx, Saccharomyces boulardii 16mxg*, *Lactobacillus acidophilus 16axg, and L. rhamnosus 18fx*, combined with the enzyme amylase based on its anti-biofilm activity [23,24,25].

In order to determine the effect of BIOHM on the comprehensive intestinal microbiome (CIM, representing bacterial and fungal communities) of human subjects, in this study, we enrolled a cohort of 49 volunteers in collaboration with the Fermentation Festival group (Santa Barbara, California). The CIM profiles of bacterial and fungal communities were assessed at baseline and following 4 weeks of BIOHM use. We then compared the bacteriome of our subjects with those reported by the Human Microbiome Project (HMP) for healthy subjects as a control for bacterial abundance. For fungal controls, we used cumulative fungal abundance data generated through the BIOHM Gut Test data repository of healthy individuals.

## 2. Materials and Methods

### 2.1. Design of BIOHM Probiotic

Appropriate probiotic strain selection is critical to the probiotic design process. To select optimal probiotic strains that antagonize (inhibit the growth of) harmful microorganisms while supporting beneficial ones, we conducted correlation analyses of bacterial–bacterial and bacterial–fungal interactions. Based on our results, we identified individual bacterial and yeast strains that antagonize *Candida* (*Lactobacillus rhamnosus 18fx* (2.38 × 10^10^ CFU/g), *Saccharomyces boulardii 16mxg* (5.6 × 10^9^ CFU/g), and *Lactobacillus acidophilus 16axg* (2.38 × 10^10^ CFU/g)), as well as a bacterium that antagonizes both *S. marcescens* and *E. coli* (*Bifidobacterium breve 19bx* (2.38 × 10^9^ CFU/g)) [26].

Based on our data, which showed that fungi and bacteria cooperate in strategic ways to form pathogenic, inflammation-inducing biofilms, we included the enzyme amylase in our formulation, which has been shown to inhibit biofilms and can be safely incorporated into a probiotic mixture [23].

Prior to reaching the small intestine, probiotics must first pass through the harsh acidic environment of the stomach. The pH of the stomach can increase to a range of 4.0–6.0 after ingestion of a meal but normally returns to the baseline acidic range of 1.5–3.5 within approximately 2 h [27]. It has been estimated that only 20–40% of probiotic cells survive this acidic exposure [28]. Previously, we evaluated the ability of selected BIOHM probiotic strains to survive at acidic conditions and showed that the *S. boulardii* and *L. rhamnosus* can survive at a pH of 1.5, while *L. acidophilus* and *B. breve* are able to survive the acidified stomach environment if ingested within 30 min of a meal [29].

### 2.2. Participants

To evaluate the effect of BIOHM on the microbiome structure of healthy individuals, we collaborated with the slow-food movement Fermentation Festival, Santa Barbara group (the slow-food movement was founded by Carlo Petrinin in 1986 as an alternative to “fast food”; proponents encourage traditional cooking of locally grown produce and livestock) to enroll in the present study [30]. Fecal samples were collected from volunteers (*n* = 49) who signed informed consent at baseline and following 4 weeks of once-a-day BIOHM consumption, these individuals are represented as “before” and “after” in all figures. In addition, a “normal” population was generated by comparing the bacteriome of our subjects to those reported by the Human Microbiome Project (HMP) for healthy subjects as a control for bacterial abundance (see below). For fungal “normal” controls, we used cumulative fungal abundance data generated through the BIOHM Gut Test data repository of healthy individuals.

### 2.3. HMP Patient Comparison Selection

To select the healthy normal subjects, we followed the inclusion and exclusion guidelines of the Human Microbiome Project [31]. Specifically, we excluded subjects that reported any chronic disease (e.g., diabetes, heart disease, overweight defined as having BMI > 35 kg/m^2^, as well as subjects on medications (especially antibiotics, antifungals, acid reflux medications, etc.). This resulted in selecting 950 individuals considered healthy (age ranges included 18–34, 34–54, 55+), with a BMI of 18.6–34.9 kg/m^2^, in our analysis.

### 2.4. DNA Extraction

Fecal samples were analyzed for their bacterial and fungal communities using Ion Torrent sequencing technology. Samples were transferred to tubes containing glass beads with the lysis solution included in the QiaAmpFast DNA Extraction Kit (QIAGEN, Germantown, MD, USA). Bacterial and fungal DNAs were isolated and purified following the manufacturer’s instructions with minor modifications: In this regard, we incorporated an additional bead-beating step (Sigma-Aldrich beads, diameter = 500 µm), with the MP FastPrep-24 speed setting of 6 M/s and 2 × 40 s cycles. The quality and purity of the isolated genomic DNA were confirmed using a NanoDrop 2000 (Fisher Scientific, Waltham, MA, USA). DNA concentration was quantified using a Qubit 2.0 instrument applying the Qubit dsDNA HS Assay (Life Technologies, Carlsbad, CA USA) and adjusted to 100 ng per sample. Extracted DNA samples were stored at −20 °C.

### 2.5. Bacterial 16S rRNA Gene or Pan Fungal ITS Amplicon Library Preparation

For bacteria, the V3-V4 region of the 16S rRNA gene was amplified using 16S-515F: GTGCCAGCMGCCGCGGTAA and 16s-806R: GGACTACHVGGGTWTCTAAT primers, while the fungal ITS region was amplified using ITS1 (CTTGGTCATTTAGAGGAAGTAA) and ITS 2 (GCTGCGTTCTTCATCGATGC) primers. The reactions were carried out on a 100 ng template DNA, in a 50 µL (final volume) reaction mixture consisting of Q5 PCR Master Mix (ThermoScientific, Waltham, MA, USA), for a final primer concentration of 400 nM. Initial denaturation at 94 °C for 3 min was followed by 30 cycles of denaturation for 30 s each at 94 °C, annealing at 57 °C (16 s) or 59 °C (ITS) for 30 s, and extension at 72 °C for 10 s. Following the 30-cycle amplification, there was a final extension time of 15 s at 72 °C. The size and quality of amplicons were screened on a 1.5% TAE agarose gel, separated using 100v, and electrophoresed for 45 min then stained with ethidium bromide. The PCR products were sheared for 20 min, using Ion Shear Plus Fragment Library Kit (Life Technologies, Carlsbad, CA, USA). The amplicon library was generated with sheared PCR products using Ion Plus Fragment Library Kit (<350 bp) according to the manufacturer’s instructions. The library was barcoded with Ion Xpress™ Barcode Adapter and ligated with the A and P1 adaptors.

### 2.6. Next-Generation Sequencing, Classification, and Analysis

The adapted barcoded libraries were concentrated 4–6× in a speed-vac (ThermoScientific, Waltham, MA, USA) and the concentrated pooled libraries were then quantified using a TaqMan Quantitation Kit (ThermoScientific, Waltham, MA, USA). The libraries were adjusted to 100 pM and attached to the surface of Ion Sphere particles (ISPs) using an Ion PGM Template OT2 400 bp Hi-Q View Kit (LifeTechnologies, Carlsbad, CA, USA) according to the manufacturer’s instructions, via emulsion PCR. The quality of ISP templates was checked using Ion Sphere™ Quality Control Kit (Part no. 4468656) with the Qubit 2.0 device. Sequencing of the pooled libraries was carried out on an Ion Torrent PGM System using the Ion Sequencing 400 bp Hi-Q View Kit (Life Technologies, Carlsbad, CA, USA) for 150 cycles (600 flows) with a 318 v2 chip, following the manufacturer’s instructions. De-multiplexing and classification were performed using the Qiime Platform (ver. 1.8). The resulting sequence data were trimmed to remove adapters, barcodes, and primers during the de-multiplexing process. In addition, the sequence data were filtered for the removal of low-quality reads below the Q25 Phred score and de-noised to exclude sequences with a read length below 100 bp [32]. De novo OTU’s were clustered using the Uclust algorithm and defined by 97% sequence similarity [33]. Classification at the species level was referenced using the Greengenes (v. 13.8) reference database [34] and taxa assigned using the nBlast method with a 90% confidence cut-off [35]. Abundance profiles for the microbiota were generated and imported into Partek Discover Suite v6.11 for principal components analysis (PCA). Diversity and correlation analyses and Kruskal–Wallis (non-parametric) analysis of variance were performed using abundance data and R statistical analysis software (CRAN, and Morgan) with packages (Psych and Vegan, Bioconductor). Diversity indices, including SDI, Richness (N), and PE, were calculated at all taxonomic levels.

### 2.7. Statistical Analyses

Pre- and post-BIOHM consumption data were analyzed for each sample. Statistical significance levels were calculated, comparing the changes across groups by *t*-test for a given genus, species, or phylum. A *p* value < 0.05 was considered significant.

## 3. Results

### 3.1. Effect of BIOHM on the Mycobiome Community

**Figure 1:**
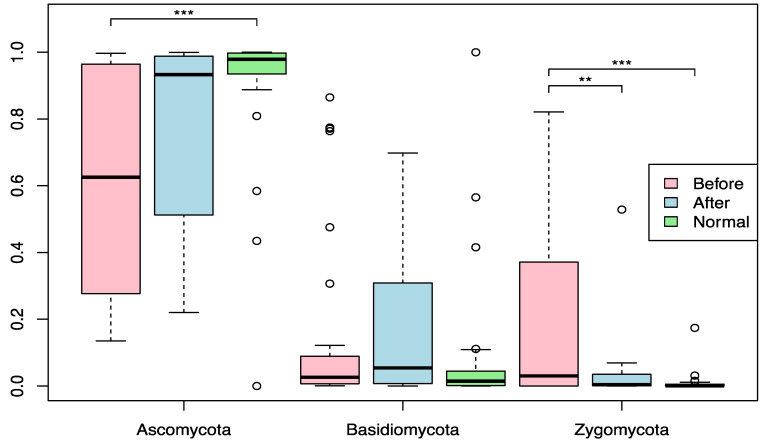
Phyla level abundance profile of the mycobiome community. Fecal samples were collected from subjects at baseline and following 4 weeks of once-a-day consumption of the probiotic BIOHM. The phyla level comparison of mycobiome abundance is shown for baseline (Before) and post-4 week consumption of BIOHM (After). Reference abundance levels (Normal) of the representative phyla are shown based upon the average abundance of a cohort of healthy individuals taken from the participants of the BIOHM gut survey (*n* = 950). *, *p* < 0.05; **, *p* < 0.01; ***, *p* < 0.001.

Figure 1 shows the phyla level profile of the mycobiome community before and after BIOHM consumption, compared with the level of fungal phyla observed in “normal” healthy individuals from the BIOHM gut testing platform cohort. Enrolled subjects had significantly lower levels of the phylum Ascomycota at baseline, compared with controls, while the level of phylum Zygomycota of the participants was significantly higher at baseline. No significant difference in Basidiomycota was observed in enrolled individuals compared to the healthy profile.

Mycobiome analysis following probiotic consumption (“after”) showed an increase in Ascomycota levels in enrolled individuals, and the abundance of this phylum increased to levels observed in healthy control profiles, a reduction in Zygomycota levels (*p* value < 0.01) with a subsequent decrease in phylum abundance also matched healthy control profiles. No statistically significant difference in Basidiomycota was detected between pre- and post-BIOHM samples and control abundance profiles (*p* > 0.05).

### 3.2. Effect of BIOHM on Candida Genus and Species Level

**Figure 2:**
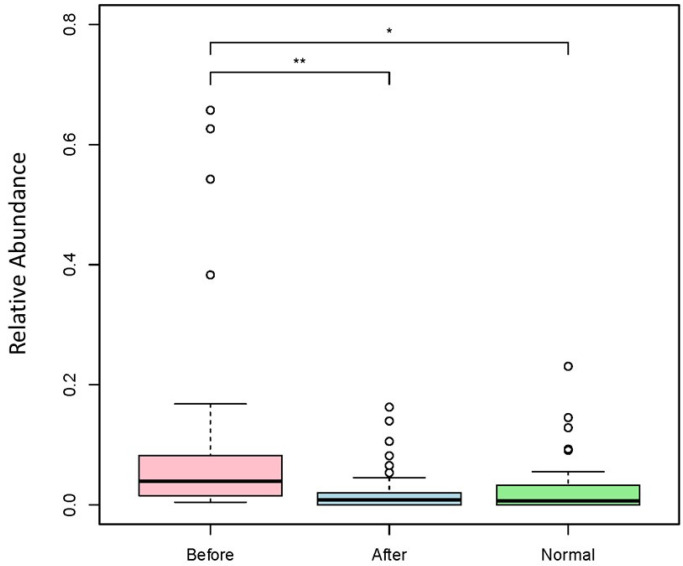
Genus level *Candida* spp. abundance levels. Fecal samples were collected from subjects at baseline and following 4 weeks of once-a-day consumption of the probiotic BIOHM. The genus-level comparison of *Candida* spp. abundance is shown for baseline (Before) and post-4 week consumption of BIOHM (After). Reference abundance levels (Normal) of *Candida* spp. were generated from the average abundance of *Candida* spp. in a cohort of healthy individuals who participated in providing samples to BIOHM for gut survey testing (*n* = 950). *, *p* < 0.05; **, *p* < 0.01; ***, *p* < 0.001.

**Figure 3:**
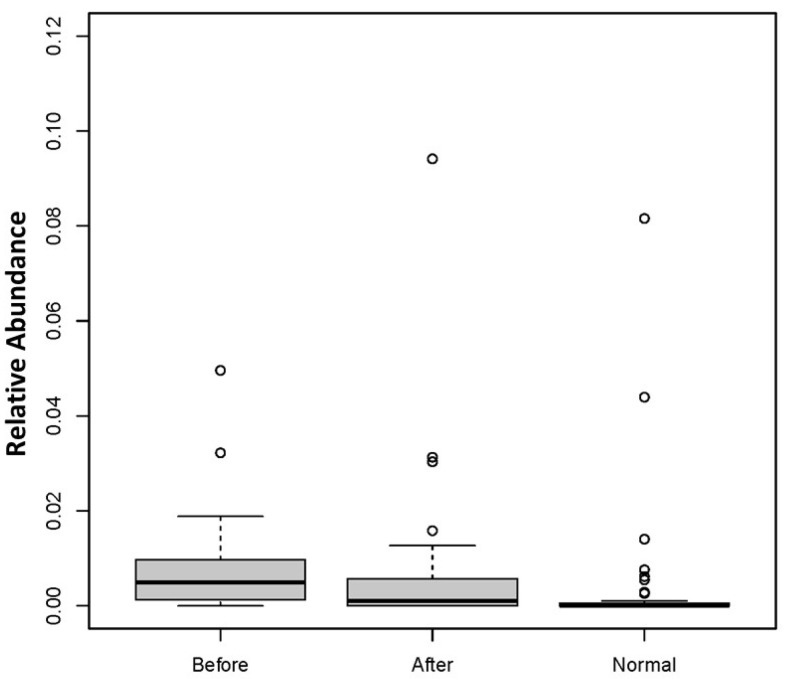
*Candida albicans* abundance levels before and after 4 weeks of BIOHM consumption. Fecal samples were collected from subjects at baseline and following 4 weeks of once-a-day consumption of the probiotic BIOHM. The *Candida albicans* abundance level is shown for baseline (Before) and post-4 week consumption of BIOHM (After). Reference abundance levels (Normal) of *Candida albicans* were generated from the average abundance of *Candida albicans* in a cohort of healthy individuals who participated in providing samples to BIOHM for gut survey testing (*n* = 950).

Abundance levels of *Candida* genus and *C. albicans* before and after BIOHM are shown in Figure 2 and Figure 3, respectively. Our data show that BIOHM consumption led to a significant reduction in the abundance of *Candida* genus in tested subjects (*p* value < 0.013), while the abundance of *C. albicans* also tended to be lower than before BIOHM use, albeit not reaching statistical significance, compared with healthy control profiles (Figure 2). The level of *C. albicans* at baseline also tended to be higher than the cumulative healthy subject average abundance (Figure 3).

### 3.3. Effect of BIOHM on the Bacteriome Community

**Figure 4:**
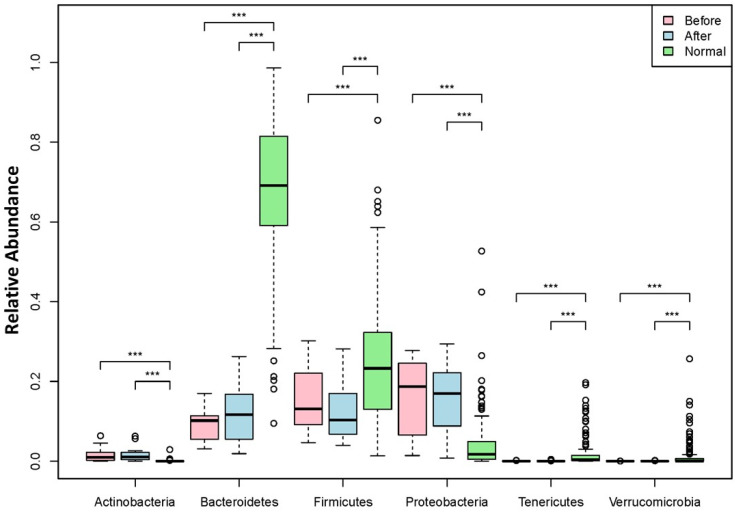
Phyla level abundance profile of the bacteriome community. Fecal samples were collected from subjects at baseline and following 4 weeks of once-a-day consumption of the probiotic BIOHM. The phyla level comparison of bacteriome abundance is shown for baseline (Before) and post-4 week consumption of BIOHM (After). Reference abundance levels (Normal) of the representative phyla are shown based upon the average abundance of these phyla in healthy control subjects who participated in the Human Microbiome Project (*n* = 250). *, *p* < 0.05; **, *p* < 0.01; ***, *p* < 0.001.

Our data showed baseline enrolled subjects had significantly lower phylum levels of Bacteroidetes, compared with the HMP healthy control cohort, while the phylum level of Firmicutes of these subjects was higher at baseline (*p* value < 0.01). Subjects in the enrolled cohort had significantly higher phylum levels of Proteobacteria (known to be a red flag for inflammation) at baseline, compared with the HMP healthy control values (*p* value < 0.001). The phyla Actinobacteria, Tenericutes, and Verrucomicrobia were detected at low abundance in all subjects irrespective of the time of collection relative to BIOHM use (Figure 4).

A reduction in the abundance of Firmicutes at the phylum level was noted following BIOHM use, which approached levels reported for HMP controls. No significant changes before and after BIOHM use were noted in the other phyla.

## 4. Discussion

Several relevant changes occurred in the GI systems of subjects in the BIOHM cohort. A 4-week regimen of a once-a-day dosage of BIOHM reduced gut dysbiosis of *Candida* at the genus level, compared with the healthy control profile. Of particular significance to our study is the reduction in *Candida* numbers in the gut. Diarrhea is a common side effect of antibiotic use associated with the treatment of IBD, due to the eradication of beneficial along with harmful bacteria. As a result, *Candida* can overgrow in the GI tract, leading to further dysbiosis. For example, *C. tropicalis*, as well as *C. albicans*, have been shown to be elevated in CD [26,36].

Beneficial changes in the bacterial community following BIOHM consumption were also demonstrated. Noteworthy was the normalization of the abundance ratio between Bacteroidetes and Firmicutes bacterial phyla. In the healthy gut, Bacteroidetes will outnumber Firmicutes strains, and a disruption of this balance may lead to obesity or sleep disorders [37,38]. Thus, the increase in Bacteroidetes and decrease in Firmicutes following BIOHM use suggested an improved balance between these strains of organisms.

Our previous work demonstrated that *C. tropicalis*, *S. marcescens*, and *E. coli* are over-abundant in CD patients, suggesting that these organisms may form a mixed-species biofilm in the gut. Data from our previously reported in vitro study demonstrated that the culture filtrate from the BIOHM probiotic strains inhibited fungal growth and germination, and possessed activity against both planktonic and biofilm forms of *Candida*, suggesting that this activity is mediated by secretory factors [2]. Given these observations, one potential strategic approach to limiting the polymicrobial interactions observed in IBD would be through the judicious use of a probiotic nutritional supplement.

Traditional approaches to IBD treatment include the use of biologic therapies such as humanized monoclonal antibodies [39] that target and block specific immune pathways that drive mucosal inflammation. Although these types of therapies have proven to be successful in inducing and maintaining remission, patients often become recalcitrant to their effects over time [40].

In an effort to circumvent the associated risks of biologic therapy, antimicrobials have also been employed to control inflammatory symptoms resulting from pathogenic bacteria and fungi colonizing the gut. However, while some patients report relief of IBD symptoms during antibiotic therapy, concerns remain with respect to tolerability, long-term safety, and the emergence of resistant strains [41]. Equally relevant to gut health is the effect of antibiotic use on the bacteriome, or bacterial makeup, of the gut microbiome. Antibiotics may have several adverse effects, which may include the development of resistant antibacterial strains, reduction in beneficial bacteria that produce vitamins such as vitamin K, lower diversity of microbial species that may lead to increased susceptibility to pathogens, and changes to immune reactions in the gut [42]. Importantly, it is becoming clear that broad-spectrum antibiotic use leads to the eradication of pathogenic bacteria as well as beneficial ones, particularly in the gut [43]. As a consequence of the antibiotic effect, *Candida* living in the GI tract overgrow, leading to further dysbiosis.

In that regard, enteric colonization by *Candida* is the most important predictor of invasive fungal infections [44]. It is important to note, however, that *Candida* colonizes the GI tract in over half of healthy individuals as well [45], and the development of mucosal or systemic candidiasis can occur due to hormonal imbalance and immunosuppressive conditions in addition to antibiotic overuse [46]. Thus, designing new strategies that enhance beneficial microbes while inhibiting the expansion of detrimental organisms is desirable.

Recently, new over-the-counter probiotic products have been developed with the goal of preventing and ameliorating gut dysbiosis and IBD. In a previous in vitro study, we determined the effect of a novel formulation containing the probiotic strains *S. boulardii*, *B. breve, L. acidophilus*, and *L. rhamnosus* on pathogenic yeast and enteric bacteria, identified as possible contributors to the inflammatory process [2].

*S. boulardii*, a well-known probiotic species, is widely used for the prevention and/or treatment of intestinal disorders, including antimicrobial-associated diarrhea, recurrent *Clostridioides difficile* (previously *Clostridium difficile*) disease, acute diarrhea in adults and children induced by a variety of enteric pathogens, traveler’s diarrhea, and relapses of CD or UC. Benefits of *S. boulardii* are believed to be related to direct enzymatic effects, modulation of the gut endogenous flora, and enhancement of the immune response. Samonis et al. evaluated the virulence of *S. boulardii* when used as a probiotic, and its role in preventing GI colonization by Candida in a murine model [47]. They showed that the gut colonization was proportional to the given dose but lasted only one week; no dissemination of the yeast was detected.

*Lactobacillus* spp., *Bifidobacterium* spp., and *S. boulardii* have shown efficacy against intestinal disorders, especially if treatment is introduced early. Orally administered *L. acidophilus* and *L. rhamnosus* (as cheese ingredients) have also been shown to reduce oral *Candida* colonization in denture wearers [48].

*An* in vitro study by Ribeiro et al. showed that both cells and supernatant of *L. rhamnosus* reduced *C. albicans* biofilm formation, filamentation, gene expression of adhesins (*ALS3* and *HWP1*), and transcriptional regulatory genes (*BCR1* and *CPH1*) [49]. Furthermore, probiotics have been described as a potential strategy to control opportunistic infections due to their ability to stimulate the immune system. In an in vivo study by Rossoni et al., strains of *L. paracasei*, *L. rhamnosus*, and *L. fermentum* were used in a *Galleria mellonella* larvae model to evaluate whether clinical isolates of *Lactobacillus* spp. are able to provide protection against *C. albicans* infection [50]. Their data demonstrated that *L. paracasei* strain 28.4 had the greatest ability to prolong the survival of larvae infected with a lethal dose of *C. albicans*, demonstrating that *Lactobacillus* can modulate the immune system.

Thus, a probiotic that will restore fungal and bacterial balance in the gut should be of enormous benefit to individuals suffering from IBD, as well as to the health of the general population. The ability of BIOHM to reduce polymicrobial biofilm formation may be an outcome of particular importance considering the pathogenesis associated with biofilms and the refractory nature of organisms incorporated in biofilms to traditional therapeutics [2]. The ability to limit biofilm formed by microbial pathogens may improve the overall ability to keep pathogenic organisms in check by decreasing the matrix of biofilms.

## 5. Conclusions

Our preliminary results show that BIOHM consumption results in the regulation of both bacterial and fungal abundance in the gut within 4 weeks of daily consumption. Importantly, the ability to significantly decrease the pathogenic genus *Candida* suggests that this probiotic should be further examined using expanded clinical trials including IBD patients, where we know imbalance in polymicrobial interactions is a key to dysbiosis and pathogenesis [1].

Limitations of the current study include the modest number of participants in the study as well as the lack of matched controls, although each participant did serve as their own control at baseline. Further limits include subject demographics and knowledge regarding potential dietary differences or the use of other potential probiotic regimens prior to participation in the current study. A more longitudinal sampling approach in future studies would provide more insight regarding the natural variability of the microbiome and how it reacts to external factors, such as changes in diet or the intake of probiotics.

Given our early success in demonstrating the ability of BIOHM to modulate the gut microbiome structure, more extensive placebo-controlled clinical trials are warranted to determine whether this novel probiotic could ameliorate or prevent symptoms in persons with IBD or gut dysbiosis. Further clinical implications regarding BIOHM consumption to consider are the face validity of being able to modulate both bacterial and fungal gut constituents. Modulation of the gut microbiome suggests that in addition to clinical approaches such as fecal microbiome transplant, it may be possible one day to tailor probiotics that would augment host microbial composition and may show efficacy as primary or adjuvant therapies for the treatment of diseases such as irritable bowel syndrome (IBS) or obesity. Indeed, the ability to modulate the microbiome through the rational design of probiotic would be useful in any number of clinical outcomes influenced by the gut microbiome, including potential immune modulation.

## Data Availability

The data that support the findings of this study are available on request from the corresponding author. The data are not publicly available owing to the privacy concerns of research participants.
